# Photoinduced electron transfer in novel CdSe–Cu_2_Se type II core–shell quantum dots†

**DOI:** 10.1039/c9ra02027f

**Published:** 2019-05-14

**Authors:** N. J. Simi, R. Vinayakan, V. V. Ison

**Affiliations:** Centre for Nano Bio Polymer Science and Technology, Department of Physics, St. Thomas College Palai, Arunapuram Kottayam-686574 Kerala India isonv@rediffmail.com +919446126926; NSS Hindu College Changanacherry Kottayam-686102 Kerala India

## Abstract

Herein we report the synthesis, characterisation and electron transfer studies of CdSe–Cu_2_Se QDs, a novel type II core–shell system. The synthesis was achieved by a high temperature organometallic method with oleylamine as ligand. Structural and optical properties of the nanostructures were investigated using X-ray diffraction, high resolution transmission electron microscopy, selected area electron diffraction, energy dispersive X-ray spectroscopy, inductive coupled plasma optical emission spectroscopy, cyclic voltammetry, X-ray photoelectron spectroscopy and absorption spectroscopy. The electron transfer dynamics were investigated by observing the variations in steady state and time resolved emission spectra in the presence of an electron acceptor-methyl viologen. Localization of electrons in the shells was evident from the studies performed indicating efficient charge separation.

## Abstract 2

Herein we report the synthesis, characterisation and electron transfer studies of CdSe–Cu_2_Se QDs, a novel type II core–shell system.

## Graphical abstract

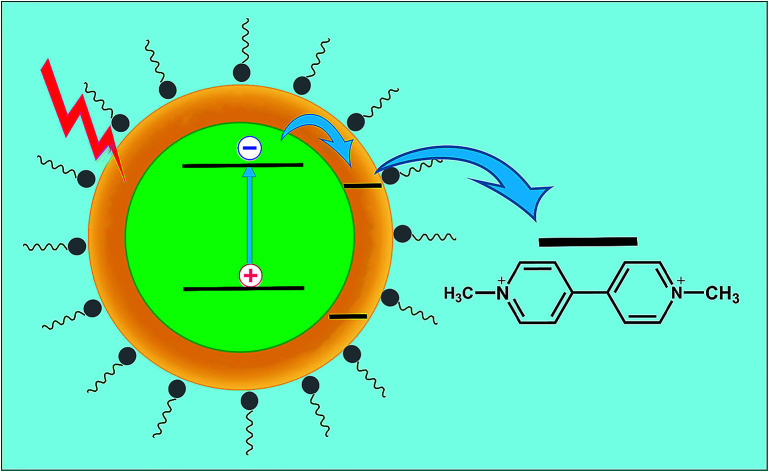

## Introduction

Core–shell quantum dots (QDs) are functional nanomaterials where ‘band engineering’ results in tailored properties that are quite different from their monocomponent counterparts.^1,2^ The possibility of adjusting the overlap of electron–hole wave functions in these systems leads to benefits in longer single^3^ and multiple^4^ exciton lifetimes, superior optical gain^5^ and reduced blinking.^6^ Their potential features make them in highly demandable in various fields such as light emitting diodes,^7^ photovoltaic devices,^8^ optical switches^9^ spintronic devices,^10^ low-threshold lasers,^11^ bio-labeling agents,^12^*etc.* Depending on the band alignment of the core and shell materials, three types of core–shell QDs systems are identified, *viz.* type I, reverse type I and type II, possessing different functional features.^1,13–16^ Among these assortments, a gifted advantage of the type II QDs is that the band gap offset in them spatially separates photo-generated carriers within the structure such that the electron-wave function resides largely in one material and the hole-wave function in the other.^6,17–20^ The energy offset can be tuned by a judicious control of the composition, size and shape of each component which offers the possibility of directly controlling the electron–hole wave function overlap, tailoring the optoelectronic properties of the devices based on them.^21^ The staggered alignment of band edges helps in improving the power conversion efficiency of photovoltaic cells by preventing the back electron transfer.^22,23^ This also causes a reduction in the oscillator strength of wave functions leading to longer lifetimes of the excited state.^9^ The exciplex state of type II QDs can raise the light absorption rate too.^23^

Different research groups reported the advancements in type II structures in consort with their characteristic features like wide absorption range extending to NIR, enhanced chemical stability, reduced recombination rate, longer exciton lifetime, efficiency enhancement in photovoltaics, *etc.*^23–28^ The band edge recombination energy of type II systems is observed to be smaller than the bandgap energy of its constituent semiconductor materials so that an emission at lower energies compared to the core (or shell) is a characteristic feature of type II QDs.^29^ A detailed investigation of the photo-induced electron/hole transfer dynamics is essential in core–shell structures, particularly while considering them as an active layer in light harvesters.^30–33^ Many research groups have used the method of monitoring the luminescence of core–shell QDs in presences of an electron donor/acceptor to investigate their charge carrier dynamics.^34,35^ Zhang *et al.* investigated the quenching of QD photoluminescence in the presence of hole acceptors and explored the static and dynamic factors involved.^36^ A similar study in core–shell nanorods by Jiang group differentiated the quenching mechanism based on the nature of binding sites. The effect of shell thickness on the charge carrier separation and recombination was also investigated in presence of electron/hole scavengers.^37^ In case of CdTe–CdSe type II core–shell system, quenching studies with anthraquinone showed an increase in charge transfer rate as well as longer exciton lifetime with an increase in thickness of CdSe shell.^38^ Again, the role of shell in charge carrier dynamics was unambiguously proved by Maity *et al.* in case of a CdS–CdTe QD-bromo-pyrogallol system.^31^

**Scheme 1:**
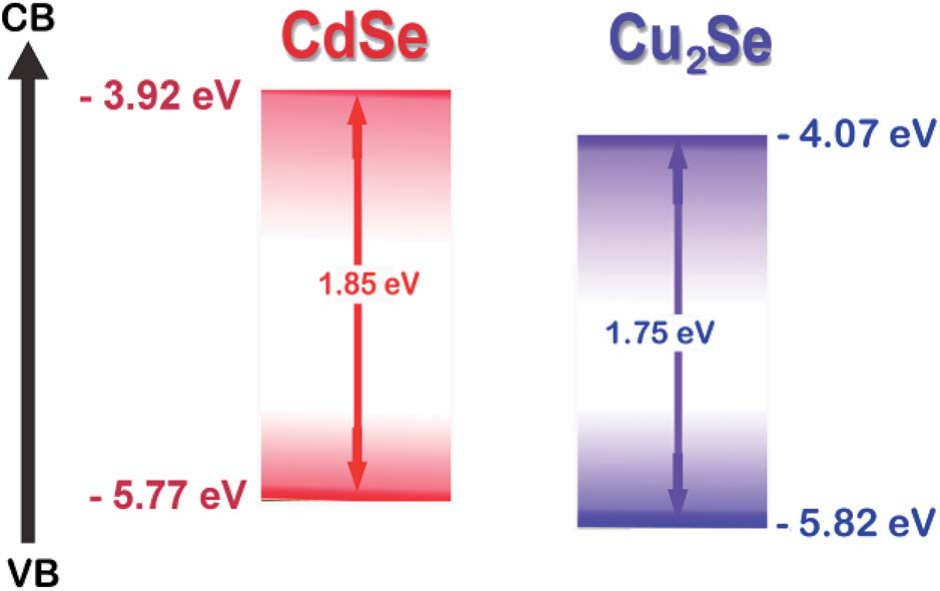
Relative band edges of CdSe and Cu_2_Se based on the CV results.

In this work, we report the synthesis of a novel CdSe–Cu_2_Se type II core–shell QDs system. Matching band alignment (Scheme 1) and minimum lattice mismatch (∼7.91%) makes Cu_2_Se an apt shell material for CdSe to generate a type II structure.^39^ Synthesis was done using organometallic high temperature route and the nanostructures were passivated by oleylamine, which is non-toxic and non-pyrophoric. The surface passivation by the amine group improved the photoluminescence quantum yield. The electron/hole transfer properties of the QDs were investigated using an electron acceptor – methyl viologen (MV^2+^) by monitoring steady state luminescence and lifetime.

## Experimental section

### Materials

Copper(i) iodide (CuI, puratronic, 99.998%) was purchased from Alfa Aesar. Cadmium oxide (CdO, 99.99%), selenium powder (Se, 200 mesh, 99.99%), oleylamine (OAm, 97%), 1-octadecene (ODE, 90%), trioctylphosphine (TOP, 90%) and methyl viologen dichloride dehydrate (98%) were received from Sigma-Aldrich. All chemicals were used as such without further purification.

### Characterisation

Absorption spectra were recorded using a Shimadzu-3600 UV-Vis-NIR spectrophotometer. Fluorescence spectra and time-correlated single photon counting (TCSPC) measurements were done with Horiba fluorolog fluorescence spectrometer equipped with 450 W Xe arc lamp (for fluorescence excitation) and 330 nm (pulse duration less than 1.5 ns) nano LED (for TCSPC). High-resolution transmission electron microscopic (HRTEM) images of the QDs were captured using JEOL 200 kV JEM-2100 microscope with Gatan Orius SC200 CCD camera after depositing them on carbon coated Cu grids. The elemental composition analysis was carried out by X-ray photoelectron spectroscopy (Shimadzu Axis Ultra X-ray photoelectron spectrometer using Mg Kα radiation), energy dispersive X-ray spectroscopy (JED-2300 analysis station) and inductive coupled plasma atomic emission spectroscopy (PerkinElmer Optima 5300 DV). The structural properties of the QDs were studied using X-ray diffractometer (Bruker AXS D8 Advance, Cu Kα radiation). Autolab 204N electrochemical workstation (METROHM AUTOLAB) with a three-electrode cell was used (scan rate 50 mV s^−1^) to record the cyclic voltammograms of the QDs. The CV analysis was done by fixing silver/silver chloride as reference electrode, glassy carbon as working electrode and platinum wire as auxiliary electrode with 0.1 M tetrabutyl ammonium hexaflurophosphate as the supporting electrolyte.

### Synthesis of CdSe QDs

Oleylamine capped CdSe QDs were synthesized by a modified version of the method reported by Peng *et al.*^40^ In brief, a mixture of CdO (16.8 mg, 0.13 mmol), OAm (3 ml, 0.009 mmol) and ODE (6 ml) was heated to 100 °C in a three necked round bottom flask under argon atmosphere with continuous stirring. The reaction mixture was degassed and kept under vacuum till the temperature was raised to 120 °C. After keeping at this temperature for 1 hour, the vacuum was replaced by argon and the heating was continued to reach 310 °C. After 30 minutes, the temperature was reduced to 300 °C and an injection mixture containing TOPSe (23.7 mg of Se in 0.6 ml TOP) was injected rapidly so that the nanocrystals growth gets initiated. After 3 minutes, the growth was arrested by cooling the reaction mixture to ambient conditions. The QDs were purified by re-precipitation with methanol three times and then with acetone. Finally, the purified QDs were dispersed in hexane for further studies.

### Synthesis of CdSe–Cu_2_Se core–shell QDs

For the synthesis, a reaction mixture of CdO (16.8 mg, 0.13 mmol), OAm (3 ml, 0.009 mmol) and ODE (6 ml) was heated to 100 °C in a three necked round bottom flask under argon atmosphere with continuous stirring. The reaction mixture was degassed and kept under vacuum till the temperature was raised to 120 °C. After keeping at this temperature for 1 hour, the vacuum was replaced with argon and the heating was continued to reach 310 °C. After 30 minutes the temperature was reduced to 300 °C and an injection mixture containing TOPSe (23.7 mg of Se in 0.6 ml TOP) was injected rapidly. After 3 minutes, CuI (13.4 mg of CuI in 0.5 ml TOP and 1 ml ODE) was introduced to effect the Cu_2_Se shell growth. The growth was arrested after desired shell growth by lowering the temperature. The QDs were purified by re-precipitation with methanol three times and then with acetone. Finally, purified QDs were dispersed in hexane for further analysis.

## Results and discussion

**Fig. 1:**
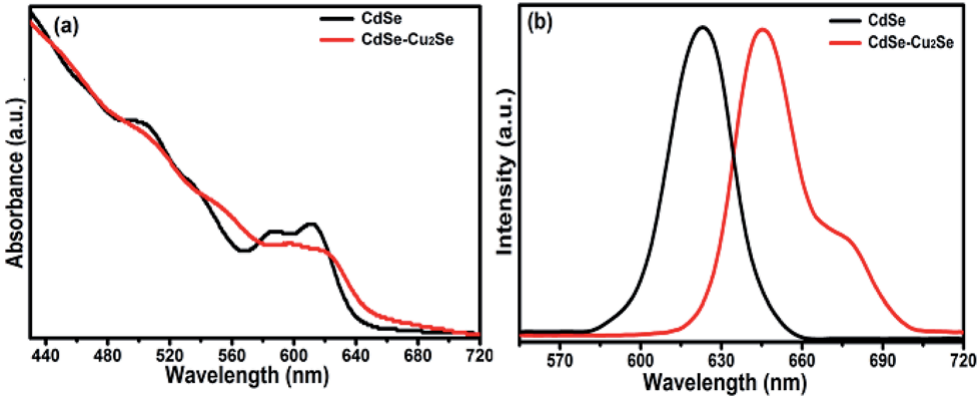
(a) Normalized UV-Vis absorption and (b) emission spectra of CdSe and CdSe–Cu_2_Se core–shell QDs.

The quantum size effects on oleylamine capped CdSe QDs was evident from the well-defined excitonic peaks (first excitonic peak at ∼611 nm) observed in the steady state absorption spectrum, recorded in hexane (Fig. 1(a)). The emission maximum was observed at ∼625 nm with a very low Stokes shift^41^ (∼14 nm), indicating that the surface passivation by oleylamine was effective in minimising surface trap states (Fig. 1(b)). Also, a low fwhm (∼25 nm) shows a narrow size distribution of the QDs. Based on the first excitonic peak value, the size of CdSe QD was estimated to be 5.1 nm.^40^ The band gap energy (*E*_g_) of CdSe QD and CdSe–Cu_2_Se QDs were determined as 1.89 and 1.80 eV respectively, by Tauc plot (Fig. S1†), using the relation 
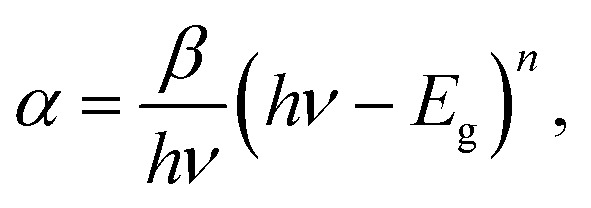
 where *α* is the linear absorption coefficient and *β* is the band gap tailoring parameter constant.^42^

As in previous reports on type II structures, the novel Cu_2_Se shell impacted considerable changes in the absorption and emission profiles of CdSe QDs (Fig. 1). A red shift in the absorption profile with disappearance of excitonic peaks was observed as a result of the Cu_2_Se shell growth (Fig. 1(a)). This is attributed to the spatially indirect type II transitions near the band edges.^43^ Decreased wave function overlap of type II QDs resulted in weaker oscillation strength causing comparatively small absorptivities near the band edge of core–shell QDs.^44^ Emission features also underwent a substantial change; a red shift in emission maximum with appearance of a shoulder peak. The intense peak was similar to that of the core QD emission while the second one was less intense and broad. This type of dual emission has already been reported in similar systems and the mechanism responsible is still under research.^45–48^ With same CdSe core size, CdSe–Cu_2_Se structures with higher shell thickness were also grown, that exhibited more or less the same features but with a nearly vanishing shoulder peak. The absorption and emission features of the bare CdSe and CdSe–Cu_2_Se core–shell QDs with different shell thicknesses are provided in the ESI (Fig. S2).† It may be noted that, the emission response is strongly affected by shell thickness. The possibility of tuning the emission response of nanostructures by different routes has already being reported by several research groups.^14,49^

**Fig. 2:**
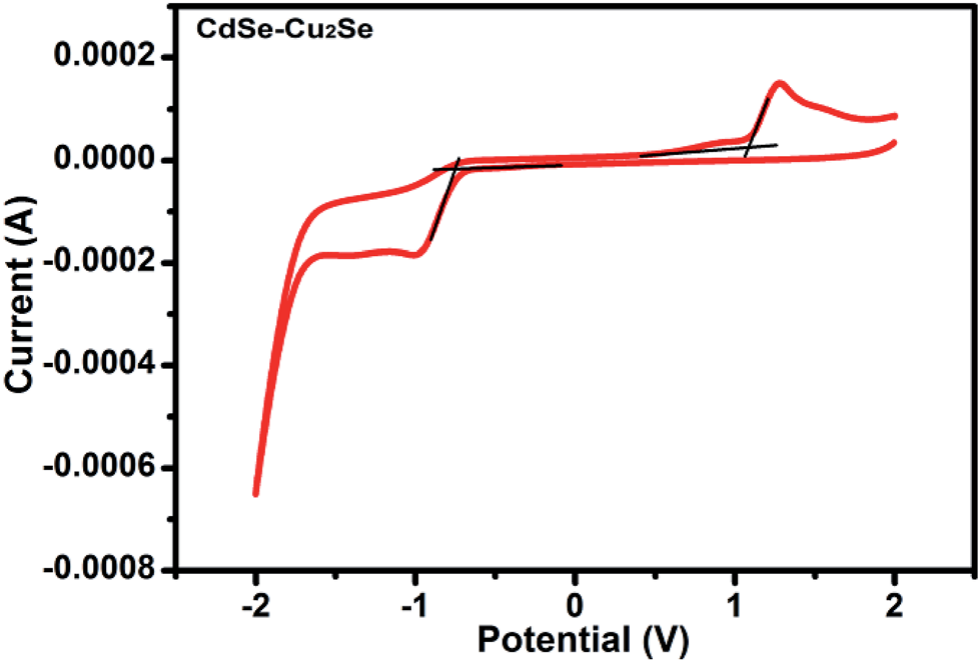
Cyclic voltammograms (CVs) of CdSe–Cu_2_Se core–shell QDs.

Band gap of the core and core–shell QDs was measured by cyclic voltammetry. The cyclic voltammograms recorded for the CdSe–Cu_2_Se core–shell QDs is provided in Fig. 2. The data for CdSe and Cu_2_Se are given in Fig. S4 of the ESI.† The band gap is calculated using the following equations.^50,51^

1 *E*_HOMO_ = −[*E*^Ox^ + 4.7] eV

2 *E*_LUMO_ = −[*E*^Red^ + 4.7] eV

The HOMO and LUMO levels were calculated from the onset of oxidation peak (*E*^Ox^) and reduction peak (*E*^Red^), which were labelled by short lines on the CV curves. The HOMO and LUMO levels of CdSe QDs were −5.78 eV and −3.90 eV respectively (Fig. S4†), from which, the band gap was calculated as 1.88 eV. This value is comparable with that obtained from the absorption peak theoretically (2.03 eV). Similarly, from the HOMO (−5.753 eV) and LUMO (−3.984 eV) energy levels, the band gap of CdSe–Cu_2_Se core–shell QDs was estimated as 1.77 eV (Fig. 2). The bandgap of CdSe and CdSe–Cu_2_Se QDs calculated using CV data are in good agreement with the values obtained from Tauc plot.

**Fig. 3:**
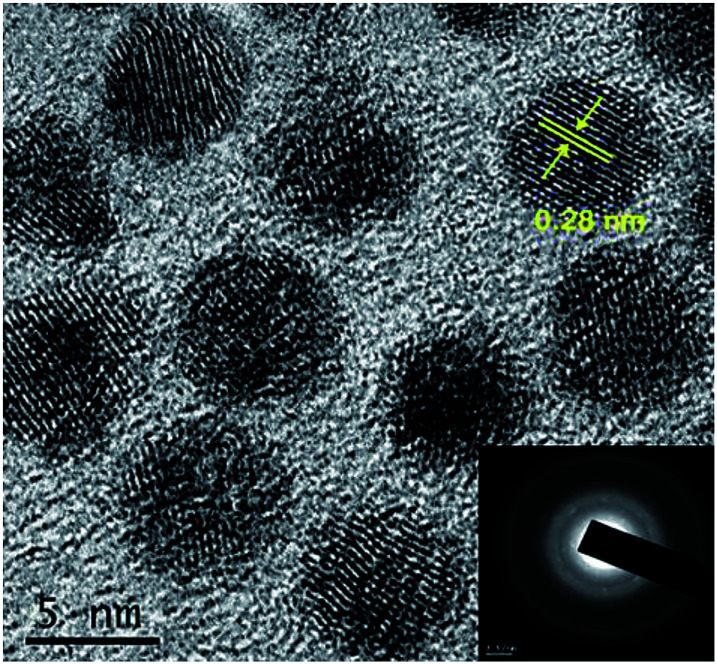
HRTEM images of CdSe–Cu_2_Se core–shell QDs. Inset shows the SAED pattern.

The size, formation of shell and crystallinity of the core and core–shell QDs were assessed by high resolution transmission electron microscope. The CdSe QDs were uniform in size distribution with an average size of 5 nm (Fig. S5†), in good agreement with that obtained from the absorption spectrum. The shell formation was evident from an increase in the overall size of the QDs, compared to core QDs, when the HRTEM images were examined (Fig. 3). The shell thickness was estimated to be 0.45 nm from TEM images in conjunction with the inductive coupled plasma-atomic emission spectroscopy (ICP-AES)^45^ and the details are given in ESI.† The boundary between the core and shell was not well clear due to matching lattice parameters of CdSe and Cu_2_Se. The lattice spacing (*d* value) was 0.28 nm corresponding to the (102) plane, in line with the d value obtained from the SAED pattern.

**Fig. 4:**
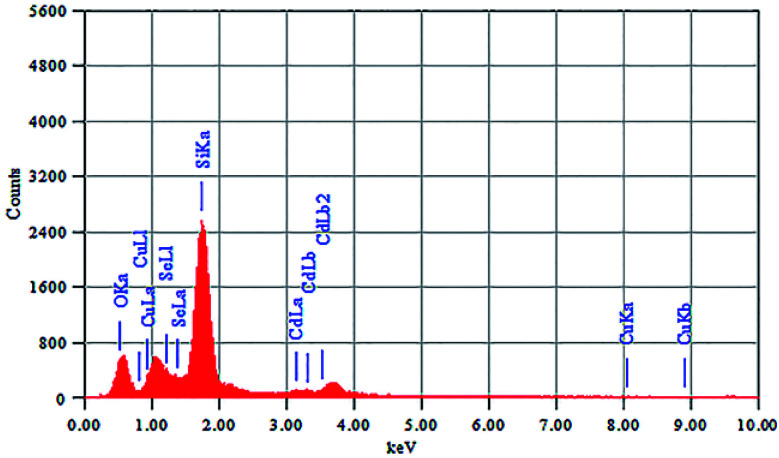
EDS spectrum of CdSe–Cu_2_Se core–shell QDs.

The elemental composition of CdSe–Cu_2_Se nanoparticles was determined using energy dispersive X-ray spectroscopy (EDS) and the spectrum is shown in Fig. 4. The corresponding data for CdSe QD is given in Fig. S6 of the ESI.† Elemental ratio of Cd, Se and Cu in the core–shell samples was obtained as 36.18 : 51.28 : 12.54 using the EDS data. The Si and O peaks were intense since the samples were coated on glass for the analysis.

**Fig. 5:**
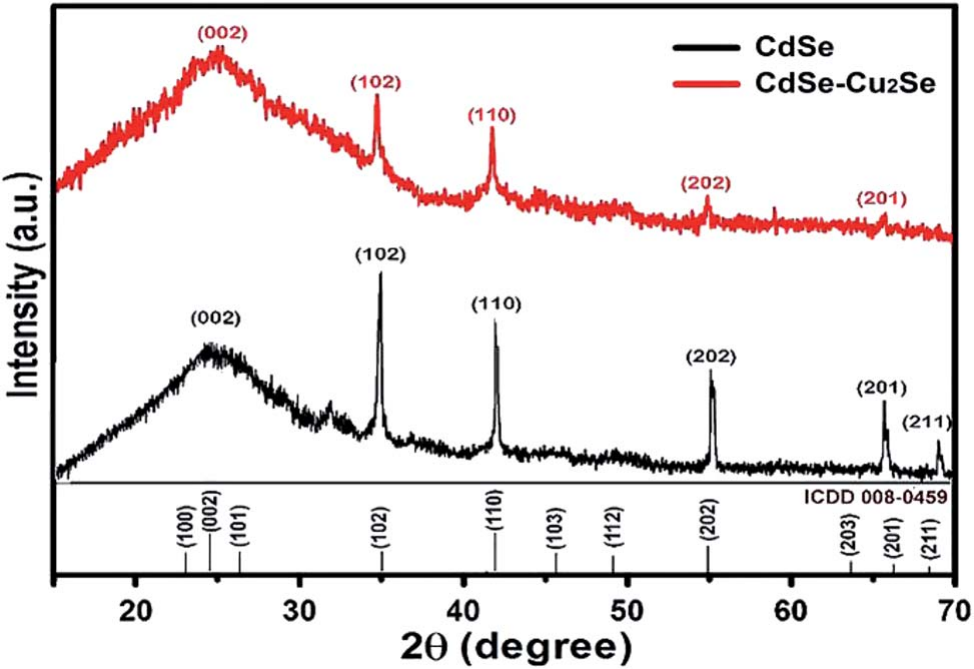
X-Ray Diffraction (XRD) patterns of CdSe and the corresponding CdSe–Cu_2_Se core–shell QDs.

X-ray diffractograms of the CdSe and CdSe–Cu_2_Se core–shell QDs are shown in Fig. 5. Nearly identical diffraction patterns are observable, confirming that both the core and the shell have similar lattice parameters. The dominant peaks at 2*θ* values, 24.4°, 35.2° and 42.1°, corresponding to the 002, 102 and 110 planes respectively, represents a hexagonal structure (ICDD card no: 01-077-2307). The size of both the core and the core–shell QDs was estimated from the XRD data using the Debye–Scherrer formula.^52,53^3
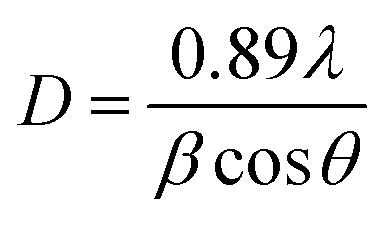
where *λ* is the wavelength of the X-ray used, *β* is the full width at half maximum (fwhm) in radians and *θ* is the Bragg angle. The size calculated for CdSe was 4.94 nm and that for CdSe–Cu_2_Se QDs was 5.86 nm respectively, in good agreement with that obtained from HRTEM.

**Fig. 6:**
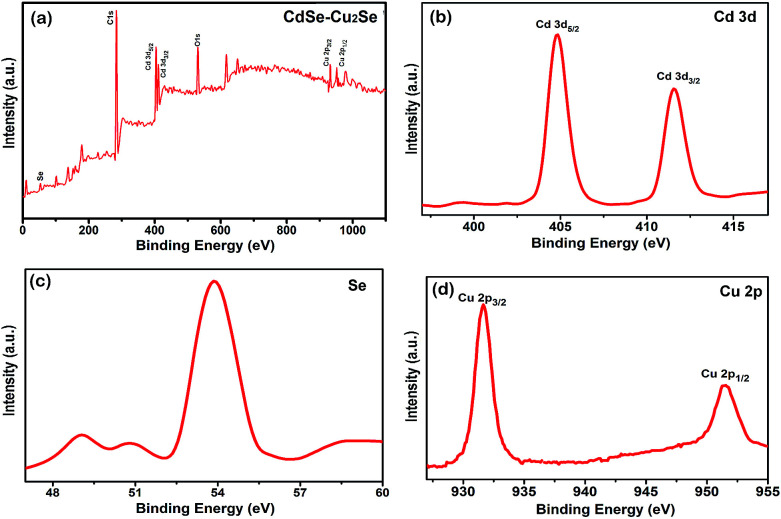
High resolution XPS spectra of (a) the core–shell QDs over the entire scan range (b) Cd 3d (c) Se 3d (d) Cu 2p.

The chemical composition and the valence state of the elements in the samples were determined by XPS analysis and result is shown in Fig. 6. The survey scan carried out showed the presence of C 1s, O 1s, Cd 3d and Se core levels in CdSe QDs (Fig. S7†). Intense C 1s and O 1s peaks were due to the exposure of the samples to air. The binding energies obtained were corrected by referring C 1s to 284.60 eV. An additional Cu 2p peak was detected in the core–shell system over the entire scan range (Fig. 6(a)) substantiating the formation of Cu_2_Se. The Cd 3d signal resulted 3d_5/2_ and 3d_3/2_ peaks (Fig. 6(b)) with a spin orbit coupling separation of 6.74 eV. The binding energies of the Cd 3d_5/2_ and 3d_3/2_ peaks of the core–shell sample (404.75 eV and 411.49 eV respectively) were in agreement with those reported in the literature.^54,55^Fig. 6(c) shows the spectrum of Se with binding energy at 53.89 eV.^56^ The Cu 2p spectrum (shown in Fig. 6(d)) has two peaks at binding energies 931.65 and 951.54 eV, separated by 19.89 eV corresponding to 2p_3/2_ and 2p_1/2_ states. The peak, characteristic of Cu^2+^ was not detected in the spectrum, confirming its monovalency.^57–59^

**Fig. 7:**
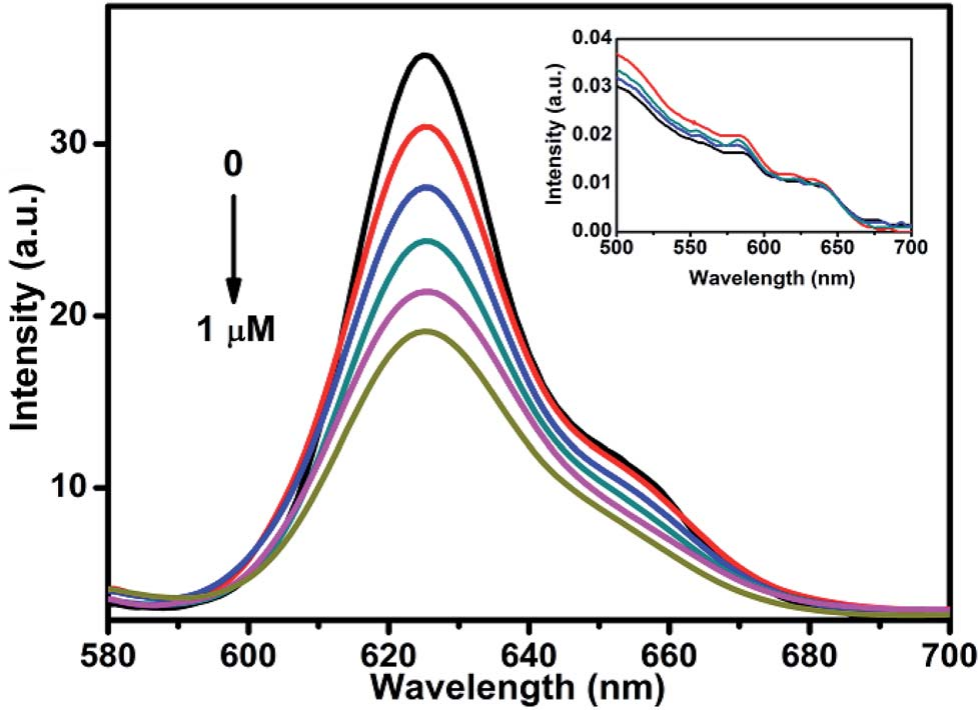
Quenching in the PL intensity of CdSe–Cu_2_Se core–shell QDs upon addition of methyl viologen (MV^2+^). (Recorded in hexane and excited at 550 nm.) Inset shows the corresponding absorption spectra.

To understand the charge carrier dynamics of CdSe–Cu_2_Se QDs, a redox reagent methyl viologen (MV^2+^) was used, and its effect on the QDs emission was examined as a function of the redox agent concentration. The relative band edge redox potential of QD and MV^2+^ (Scheme S1†) is indicative of the capability of the latter as an effective electron acceptor in our case. The change in photoluminescence intensity of CdSe–Cu_2_Se QDs in presence of MV^2+^ (1% methanol–hexane mixture) is shown in Fig. 7. A noticeable decrease in luminescence intensity was observed upon successive (0.2 μM) addition of MV^2+^. Since MV^2+^ has a lower redox level compared to the QDs, the luminescence quenching could be attributed to the scavenging of electrons from the conduction band. Since the absorption spectrum remained unaltered in presence of the quencher (inset of Fig. 7), one can rule out any ground state interaction that might otherwise have caused a change in the electronic structure of QDs while introducing MV^2+^. Besides, any chance of energy transfer between the QD and quencher can also be ruled out since the QDs are excited exclusively and there is no overlap between QD emission and the MV^2+^ absorption.^60^

**Fig. 8:**
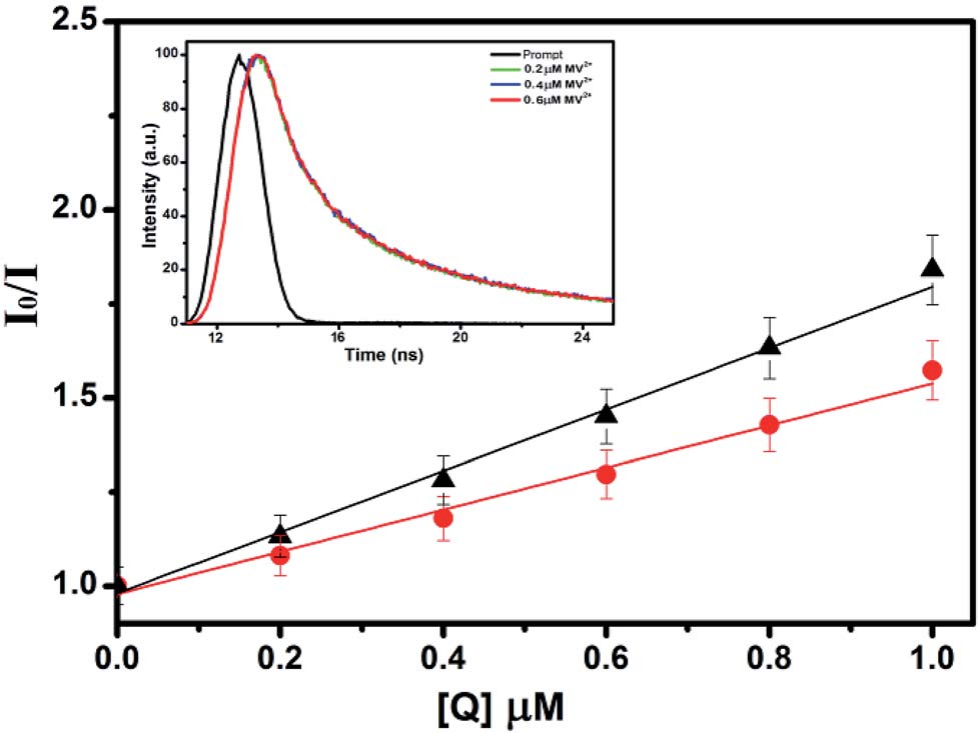
Stern–Volmer plots for comparing PL quenching of CdSe (▲) and CdSe–Cu_2_Se core–shell (●) QDs in presence of MV^2+^. Inset shows the corresponding TCSPC data (excited at 330 nm), showing little change in average lifetime.

To study the quenching efficiency of the core and the core–shell QDs, we resorted to Stern–Volmer plot which is shown in Fig. 8. Since a linear trend with a positive slope be due to static or a dynamic interaction between the QDs and the quencher molecules, we carried out a quantitative analysis of the plots to identify the mechanism involved, using the relation4
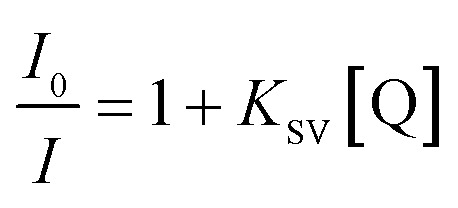
where, *I*_0_ and *I* are the luminescence intensities in presence and absence of the quencher, *Q* is the molar concentration of quencher and *K*_SV_ is the Stern–Volmer constant.^61,62^ A higher value of *K*_SV_ obtained for CdSe–Cu_2_Se core–shell QDs (8.39 × 10^5^ M^−1^) as compared to the CdSe QDs (5.57 × 10^5^ M^−1^) indicated a favourable situation in the core–shell system for charge transfer due to electron delocalization.

Further, the bimolecular quenching rate constant (*K*_q_) was also calculated for the core and the core–shell systems using the equation5
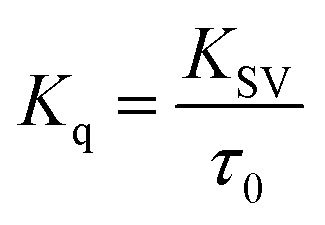
where, *τ*_0_ is the average lifetime of the core–shell QDs in the absence of the quencher. From the time correlated single photon counting analysis (TCSPC) given in the inset of Fig. 8, *τ*_0_ was estimated to be 21 ns and the corresponding *K*_q_ was found to be 3.99 × 10^13^ M^−1^ s^−1^. Since the limiting value of *K*_q_ for a dynamic interaction between a donor and an acceptor is 2 × 10^10^ M^−1^ s^−1^, a static interaction could be assigned between the core–shell QDs and MV^2+^ in this study which is also supported by the TCSPC data where there was no change of average life time of QD in presence of the quencher (detailed data provided in the ESI†).^62,63^ The same behaviour was also observed in CdSe QDs, where the *K*_q_ value obtained is 2.65 × 10^13^ M^−1^ s^−1^ indicating a static interaction.

## Conclusions

In summary, synthesis and characterisation of a novel type II core–shell QD system – CdSe–Cu_2_Se QDs was carried out. The high temperature organometallic synthesis route yielded highly crystalline, monodisperse QDs. The core–shell structure was indirectly evident from absorption and emission spectra. Energy dispersive X-ray spectroscopy and X-ray photoelectron spectroscopy also contributed in ratifying the core–shell structure. HRTEM images confirmed a uniform size distribution as well as a crystalline structure. Lattice parameters corresponding to a hexagonal structure were revealed from the XRD pattern. The envisaged electron delocalisation to shell in CdSe–Cu_2_Se type II structure was proved by an electron transfer study with methyl viologen. An associative interaction of MV^2+^ with QD was conformed from the Stern–Volmer plot analysis and the life time studies. The core–shell QDs were found to be more effective in transferring electrons to MV^2+^ as compared to their core analogue. The efficacy in charge separation in the novel CdSe–Cu_2_Se core–shell system make it a good choice while designing light harvesting devices. Detailed investigations are progressing in this direction.

## Conflicts of interest

There are no conflicts to declare.

## Supplementary Material

RA-009-C9RA02027F-s001
