# Supplementary material for: Photoinduced electron transfer in novel CdSe–Cu2Se type II core–shell quantum dots
Source: RSC Adv. 2019 May 14;9(26):15092–8. doi: 10.1039/c9ra02027f (PMC9064209; doi:10.1039/c9ra02027f)
Supplement: RA-009-C9RA02027F-s001 [file RA-009-C9RA02027F-s001.pdf]

Electronic Supplementary Material (ESI) for RSC Advances.
This journal is © The Royal Society of Chemistry 2019

Supplementary Material

1. Tauc plots of CdSe and CdSe-Cu_2Se core-shell QDs

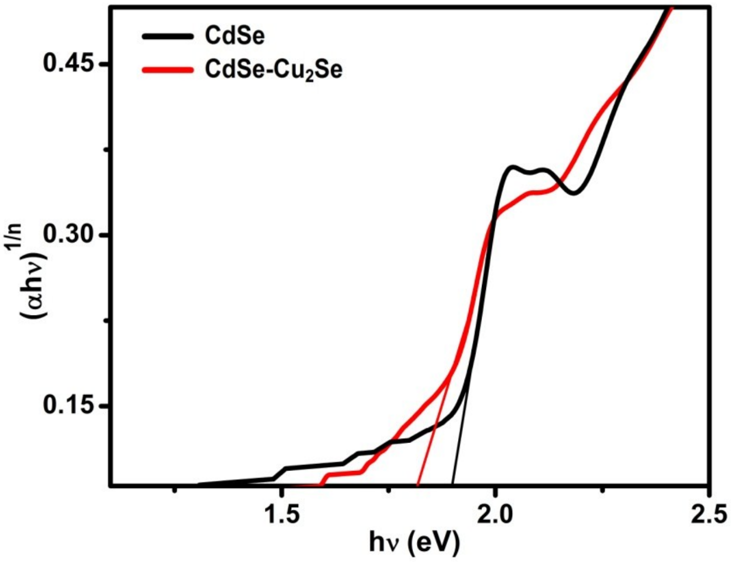

Fig. S1. Tauc plots for CdSe QD and CdSe-Cu_2Se QDs. Term 'n' take values ½ and 2 for CdSe (direct band gap) and CdSe-Cu_2Se (indirect band gap) respectively.

2. Absorption and Emission spectra of CdSe and CdSe-Cu_2Se core-shell QDs

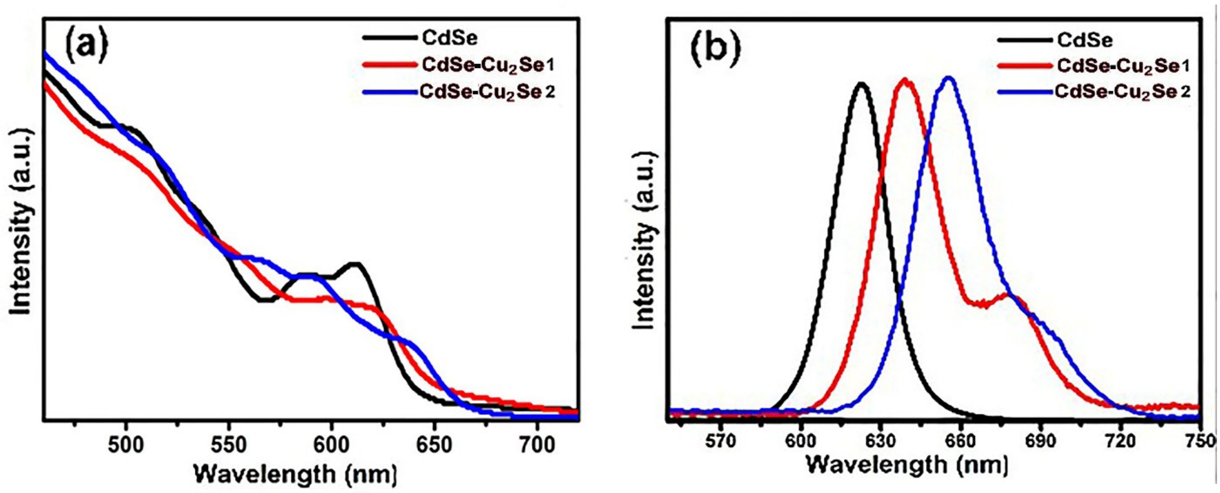

Fig. S2. Absorption and emission features of the CdSe QDs and CdSe-Cu_2Se core-shell QDs of different shell thicknesses.

3. Gaussian fitting to the emission spectra of CdSe-Cu_2Se core-shell QDs

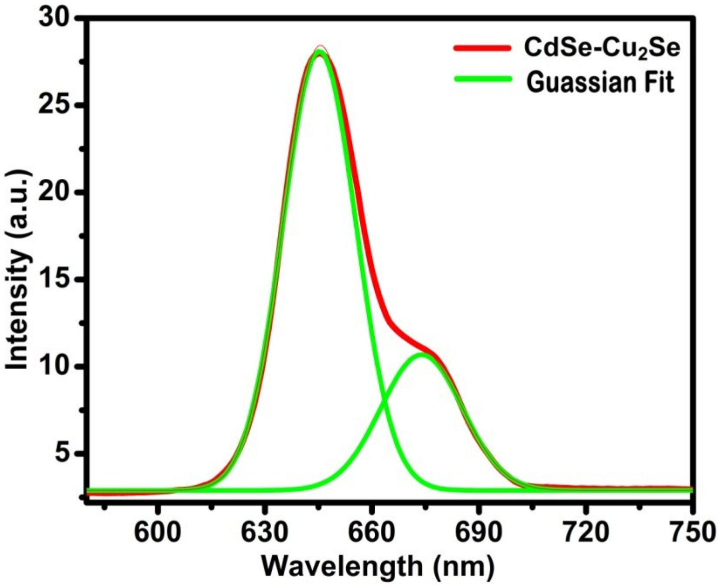

Fig. S3. PL spectrum CdSe-Cu_2Se core-shell QDs fitted with a Gaussian curve.

Table S1: Gaussian fit details to the PL spectrum of CdSe-Cu_2Se QDs

Peak	Area (nm^2)	Center (nm)	Width (nm)	Height
1	6487551.04	645.26	20.54	252006.61
2	2234293.15	673.93	22.89	77870.41

4. Cyclic Voltammograms of CdSe and Cu_2Se QDs

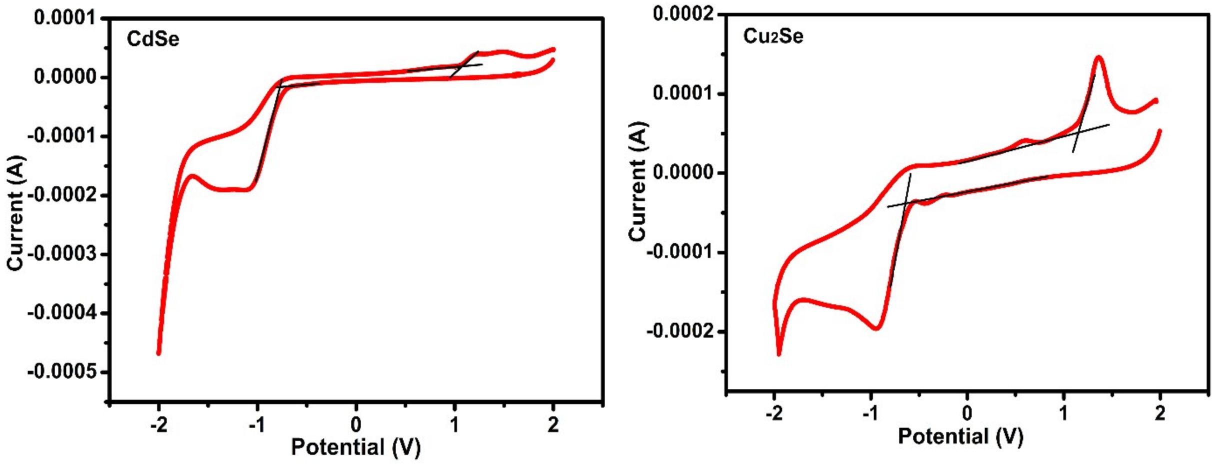

Fig. S4. Cyclic voltammograms (CVs) of CdSe and Cu_2Se QDs.

5. TEM image and SAED pattern of CdSe QDs

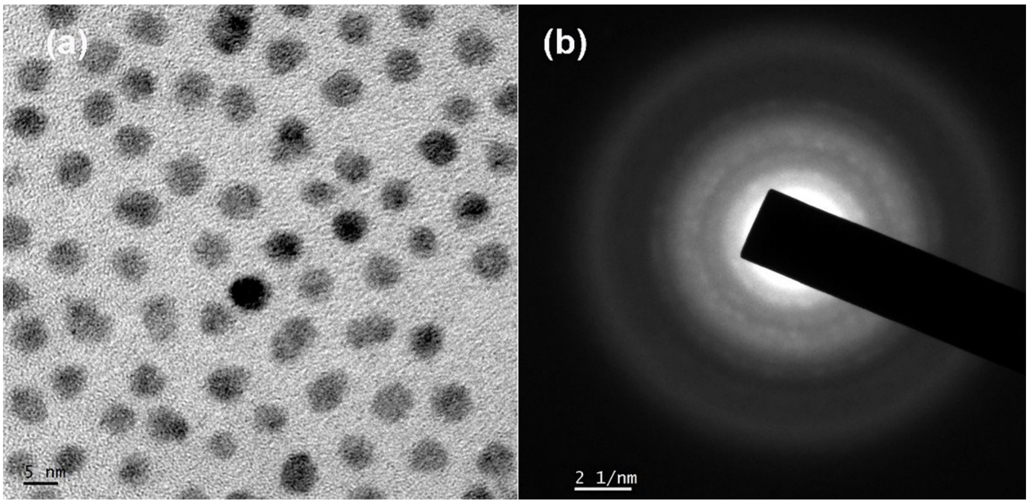

Fig. S5: TEM image of (a) CdSe QDs and (b) its SAED pattern.

6. Shell thickness calculation

The appraisal of shell thickness of CdSe-Cu_2Se QDs was done using inductively coupled plasma atomic emission spectroscopy (ICP-AES) and high-resolution transmission electron microscopy (HR-TEM) results.

The calculation was done using the following equation

$$r = \frac{R}{\sqrt[3]{1 + \frac{V_{Cu_2Se}}{V_{CdSe}}}} \quad (1)$$

where,

r is the radius of the core

R is the radius of core-shell QD

V_{CdSe} is the volume of the core

V_{Cu_2Se} is the volume of the shell

We got the radius *R* of core-shell QDs from TEM images (2.95 nm). The ratio V_{CdSe} / V_{Cu_2Se} can be calculated using the atomic and the molecular masses M_x, bulk densities ρ_x and mass ratio $\frac{m_{Cd}}{m_{Cu}}$ (obtained from ICPAES results) based on the following relation

$$\frac{V_{CdSe}}{V_{Cu_2Se}} = \frac{m_{Cd}}{m_{Cu}} \times \frac{M_{Cu}}{M_{Cd}} \times \frac{M_{CdSe}}{M_{Cu_2Se}} \times \frac{\rho_{Cu_2Se}}{\rho_{CdSe}} \quad (2)$$

$$= 0.6171 \times \frac{m_{Cd}}{m_{Cu}}$$

From ICPAES studies, we got

$$\frac{m_{Cd}}{m_{Cu}} = \frac{29.17}{11.53} = 2.53$$

Therefore, $\frac{V_{CdSe}}{V_{Cu_2Se}} = 1.56$

Substituting these results in equation (1), we get

Radius of the core *r* = 2.50 nm

Shell thickness *H* = 0.45 nm.

Radius of CdSe core obtained from TEM images was 2.55 nm. The estimated value matches with the experimental result.

7. EDS spectrum of CdSe QDs

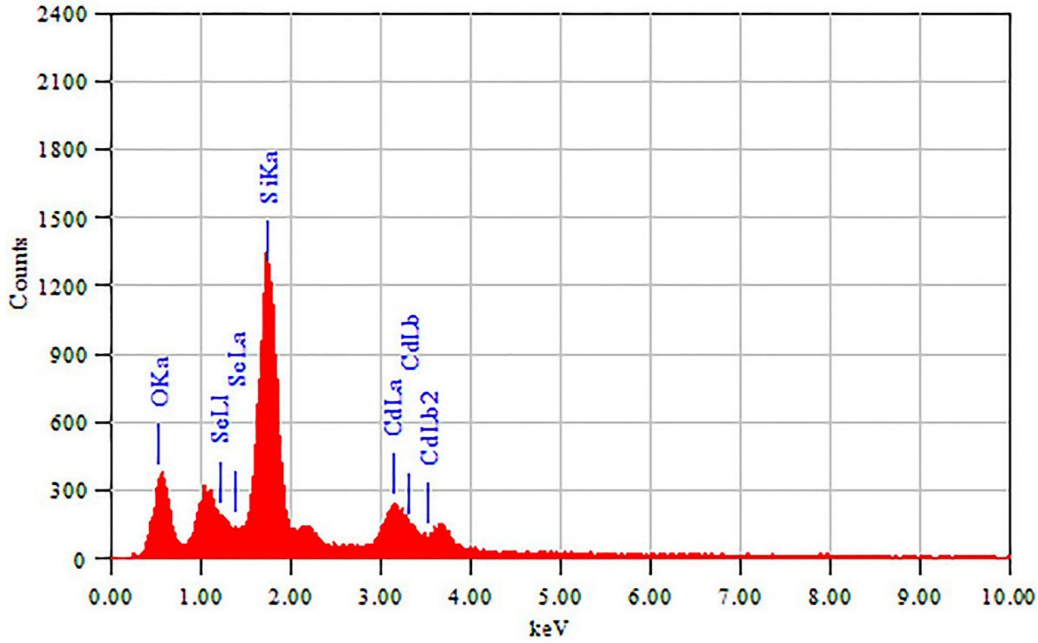

Fig. S6: EDS spectrum of CdSe QDs.

8. XPS spectrum of CdSe QDs

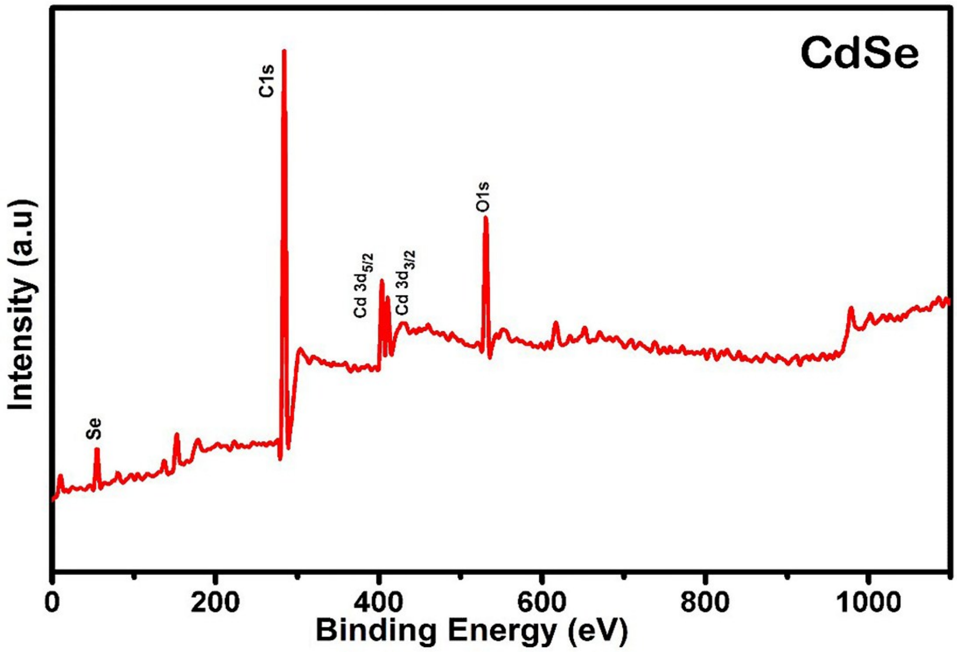

Fig. S7: XPS Spectrum of CdSe QDs.

9. Schematic representation

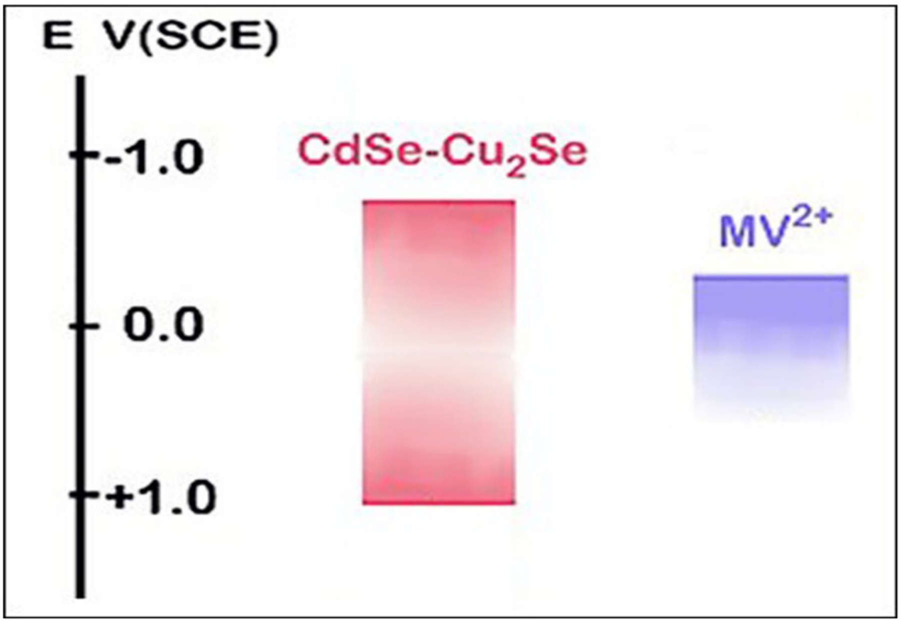

Scheme S1: Schematic picture showing the thermodynamic favorability of the electron transfer between QDs andMV^{2+}.

10. Lifetime calculations

The emission decay kinetics for CdSe and CdSe-Cu_2Se fitted multi-exponentially (3rd order) using the equation

$$\tau_{avg} = \frac{a_1\tau_1^2 + a_2\tau_2^2 + a_3\tau_3^2}{a_1\tau_1 + a_2\tau_2 + a_3\tau_3} \qquad (3)$$

Table S2: Average lifetime of bare CdSe and overcoated CdSe-Cu2Se QDs

Sample	τ_1 ,ns	a_1	τ_2 ,ns	a_2	τ_3 ,ns	a_3	τ_{avg} ,ns	χ^2
CdSe	0.285	27.56	3.792	40.08	17.68	32.36	14	1.267039
CdSe-Cu_2Se	0.835	18.18	6.58	34.41	34.23	47.41	30	1.204785

Table S3: Average lifetime of CdSe-Cu_2Se QDs/MV^{2+} complex upon successive additions (0.2 µM) of MV^{2+}

Sample	τ_1 ,ns	a_1	τ_2 ,ns	a_2	τ_3 ,ns	a_3	τ_4 ,ns	a_4	τ_{avg} ,ns	χ^2
QDs/0.2 µM MV^{2+}	0.419	36.83	3.05	37.33	9.94	9.74	39.0	16.13	21	1.185496
QDs/0.4 µM MV^{2+}	0.380	15.32	2.92	34.59	9.40	37.77	34.52	12.32	20	1.060308
QDs/0.6 µM MV^{2+}	0.430	16.09	3.05	35.38	9.59	37.30	35.88	11.23	20	1.067175
